# Field application of silicon alleviates drought stress and improves water use efficiency in wheat

**DOI:** 10.3389/fpls.2022.1030620

**Published:** 2022-11-09

**Authors:** Scott N. Johnson, Zhong-Hua Chen, Rhiannon C. Rowe, David T. Tissue

**Affiliations:** ^1^ Hawkesbury Institute for the Environment, Western Sydney University, Penrith, NSW, Australia; ^2^ School of Science, Western Sydney University, Penrith, NSW, Australia; ^3^ Global Centre for Land-Based Innovation, Western Sydney University, Richmond, NSW, Australia

**Keywords:** carbon capture, cereals, climate change, drought stress, silica, water deficits, *Triticum aestivum* L.

## Abstract

Detrimental impacts of drought on crop yield have tripled in the last 50 years with climate models predicting that the frequency of such droughts will intensify in the future. Silicon (Si) accumulation, especially in Poaceae crops such as wheat (*Triticum aestivum* L.), may alleviate the adverse impacts of drought. We have very limited information, however, about whether Si supplementation could alleviate the impacts of drought under field conditions and no studies have specifically manipulated rainfall. Using field–based rain exclusion shelters, we determined whether Si supplementation (equivalent to 39, 78 and 117 kg ha^-1^) affected *T. aestivum* growth, elemental chemistry [Si, carbon (C) and nitrogen (N)], physiology (rates of photosynthesis, transpiration, stomatal conductance, and water use efficiency) and yield (grain production) under ambient and drought (50% of ambient) rainfall scenarios. Averaged across Si treatments, drought reduced shoot mass by 21% and grain production by 18%. Si supplementation increased shoot mass by up to 43% and 73% in ambient and drought water treatments, respectively, and restored grain production in droughted plants to levels comparable with plants supplied with ambient rainfall. Si supplementation increased leaf-level water use efficiency by 32–74%, depending on Si supplementation rates. Water supply and Si supplementation did not alter *concentrations* of C and N, but Si supplementation increased shoot C *content* by 39% and 83% under ambient and drought conditions, respectively. This equates to an increase from 6.4 to 8.9 tonnes C ha^-1^ and from 4.03 to 7.35 tonnes C ha^-1^ under ambient and drought conditions, respectively. We conclude that Si supplementation ameliorated the negative impacts of drought on *T. aestivum* growth and grain yield, potentially through its beneficial impacts on water use efficiency. Moreover, the beneficial impacts of Si on plant growth and C storage may render Si supplementation a useful tool for both drought mitigation and C sequestration.

## Introduction

Global climate change models consistently predict more variable and extreme precipitation regimes in many regions, including longer and more frequent periods of drought (Easterling et al., 2000; Knapp et al., 2008; IPCC, 2013). For instance, in 2022 Europe experienced its worst drought in at least 500 years, with hot and dry conditions fuelling bushfires, impairing electricity generation, and reducing crop yields (Toreti et al., 2022). Prolonged reductions in rainfall impose significant stresses on many crops, including reduced leaf water potential, rates of photosynthesis and resource capture (Farooq et al., 2012). Reduced nitrogen (N) and phosphorus (P) concentrations in tissues are commonly observed; in a meta-analysis of 36 plant species, N and P concentrations declined by 3.7 and 9.2%, respectively (He and Dijkstra, 2014). This is usually attributed to reduced N and P uptake under drought conditions because of reduced stomatal conductance, photosynthesis and transpiration rates (Farooq et al., 2012). The severity of droughts on crop production has approximately tripled in the last 50 years with cereal crops, in particular, seeing an average yield reduction of 9% due to droughts (Brás et al., 2021). In a quantitative analysis of studies published between 1980 to 2015, Daryanto et al. (2016) reported global grain yield losses up to 21% in wheat (*Triticum aestivum* L.) and 39% in maize (*Zea mays* L.) due to drought.

With growing global populations, there is strong imperative to reduce such crop losses through mitigation measures, including new and more sustainable agricultural practices (Gregory et al., 2009). There is now widespread consensus that silicon (Si) accumulation in many plants, particularly the Poaceae, can improve tolerance to a broad range of biotic (e.g. pests and pathogens) and abiotic (e.g. drought and salinity) environmental stresses (Cooke and Leishman, 2016; Debona et al., 2017). This realisation has generated considerable interest in whether these beneficial effects could be exploited in crop production, particularly in terms of increasing Si supply and accumulation in crops (Guntzer et al., 2012). While Si is the most abundant metalloid in the soil (28%) only a small fraction, orthosilicic acid (H_4_SiO_4_), is available for plant uptake, which is deposited in and around disparate tissues (Epstein, 1999). There are numerous reports of Si supplementation alleviating the adverse effects of drought; however, the exact mechanisms are debated and vary between plant taxa and genotype (Thorne et al., 2020). Various mechanisms have been proposed including increased production of antioxidants, binding and co-precipitation with metal ions, modification of element uptake, higher hydraulic conductance and reduced water loss at the leaf surface (e.g. reduced transpiration) due to silicification (Liang et al., 2007; Cooke and Leishman, 2011). The relative importance of these physiological processes in alleviating drought stress continues to occupy researchers in this area (Thorne et al., 2020; Malik et al., 2021).

In pursuing a mechanistic understanding of Si alleviation of drought stress, most empirical work has adopted a reductionist approach using glasshouse/chamber experiments comprising pot or hydroponic systems (Thorne et al., 2020), the latter imposing osmotic stress as a surrogate for drought stress in the plant (e.g. Vandegeer et al., 2021a). Investigations of whether Si supplementation alleviates drought stress under field conditions is surprisingly rare. In wheat (*T. aestivum*), for example, there are only two studies to our knowledge (Maghsoudi et al., 2016; Schaller et al., 2021). These studies showed that Si supplementation generally had beneficial effects on wheat growing under drought conditions, including increased water use efficiency (Maghsoudi et al., 2016) and photosynthetic activity (Schaller et al., 2021). Both field studies supplemented naturally dry field sites with irrigation but did not manipulate exposure to rainfall. An important consideration in the context of climate change research, however, is to experimentally manipulate rainfall in the field to regulate how much water is available to plants, which is usually done using rain–exclusion shelters (Foale et al., 1986). This is important in the context of Si research because there is conflicting evidence whether water deficits increase (e.g. Quigley et al., 2020) or decrease (e.g. Ryalls et al., 2018) Si uptake in plants. Si uptake is driven by energy-demanding active uptake and passive uptake, with the latter being strongly influenced by the transpiration stream (Ma and Yamaji, 2015; Deshmukh and Bélanger, 2016). Since the transpiration stream is often affected by drought (Farooq et al., 2012), these conditions clearly have the potential to impact passive Si uptake.

The objective of this study was to investigate the extent to which drought adversely affects wheat growth and productivity, and whether Si supplementation under field conditions ameliorated these impacts. We used rain–exclusion shelters to manipulate the supply of rainfall and quantified changes in plant growth, elemental chemistry (Si, C and N), physiology (rates of photosynthesis, transpiration, stomatal conductance, and water use efficiency) and yield (grain production) in response to Si supplementation and water supply. We hypothesised that drought reduces plant growth, Si uptake, N concentrations, physiological processes, and yield, but Si supplementation partially or completely ameliorates these responses.

## Methods and materials

### Field site and soil preparation

The field site was located on the Hawkesbury campus of Western Sydney University. The experimental platform comprises 12 rain-exclusion shelters (249 × 166 cm) described fully by Johnson et al. (2016), but modified to have four soil-embedded rectangular pots (27 cm × 26 cm and 45 cm deep, 27 L volume) underneath each shelter in a 2 × 2 configuration. Pots were filled to a depth of 30 cm with sieved (5 mm) loam soil from the Yarramundi NSW region (–33.61, 150.74) (see **Table S1** for details). The upper 15 cm was filled with the same soil treated with mono-ammonium phosphate fertiliser (Incitec Pivot) and urea (Incitec Pivot) at an application rate of 100 and 40 kg ha^-1^, respectively (1.87 g and 0.75 g per pot, respectively) prior to planting seed. The urea treatment was repeated at the same application rates during tillering stage and boot stage of plant growth. Application rates were determined using supplier recommendations for this cultivar, described below (AGT, 2022), and nutrient levels of the soil (**Table S1**). Si treatments (see below) were premixed with the mono-ammonium phosphate and urea and incorporated into the upper soil layer by applying granules and homogenising the soil. For disease control, plants were monitored for disease and were initially treated with Folicur according to manufacturer’s recommendations (Bayer, NSW, Australia), as required.

### Experimental design

#### Silicon treatments

For each shelter, the upper 15 cm (c. 23.5 kg) of soil was either not supplemented with Si (control) or treated with one of three levels of amorphous silica (Agrisilica, Agripower, NSW, Australia), which is a form of diatomaceous earth, comprising the diatom (*Melosira granulata*) (Minimum values: 26% Si; 2.2% Calcium; 1.2% Magnesium; 2.1% Iron). The three treatments represent field application of Agrisilica at the rate of 150, 300 and 450 kg ha^-1^ (Si supplementation of 39, 78 and 117 kg ha^-1^) which were achieved by adding 2.81, 5.62 and 8.43 g of Agrisilica, respectively, to each pot. Application rates for all fertilization followed the procedure described by Imakumbili (2019).

#### Planting and irrigation regimes

The wheat (*Triticum aestivum*) cultivar Sunflex was supplied by Australian Grain Technologies (AGT, SA, Australia). Sunflex is an early season cultivar sown in April–May (Autumn) and suited to regions in Australia receiving moderate–high rainfall (AGT, 2022). Ten equally spaced seeds were initially planted in each pot on 11 May 2021 and pots watered to field capacity. Established seedlings were removed three days later so that each pot had five plants, which equates to a sowing density of 71 plants per m^2^. Half of the six replicate pots were fitted with Time Domain Reflectometry (TDR) soil moisture probes (CS616, Campbell Scientific, Thuringowa, QLD, Australia) with 30 cm long prongs installed at an angle of 30° to monitor soil moisture levels in the top 15 cm of the soil profile. Regular (approximately every 5 weeks) measurements of soil moisture were also conducted manually in all plots using a theta probe [Delta T Devices, Cambridge, United Kingdom (UK)] to verify automatically logged moisture readings (Power et al., 2016). All plants were maintained at 80% field capacity for the first 50 days post full emergence (10 days after planting) to ensure plant establishment. The soil water content (SWC) at field capacity was determined using five additional pots of soil (without plants) as previously described in Vandegeer et al. (2020).

**Figure 1:**
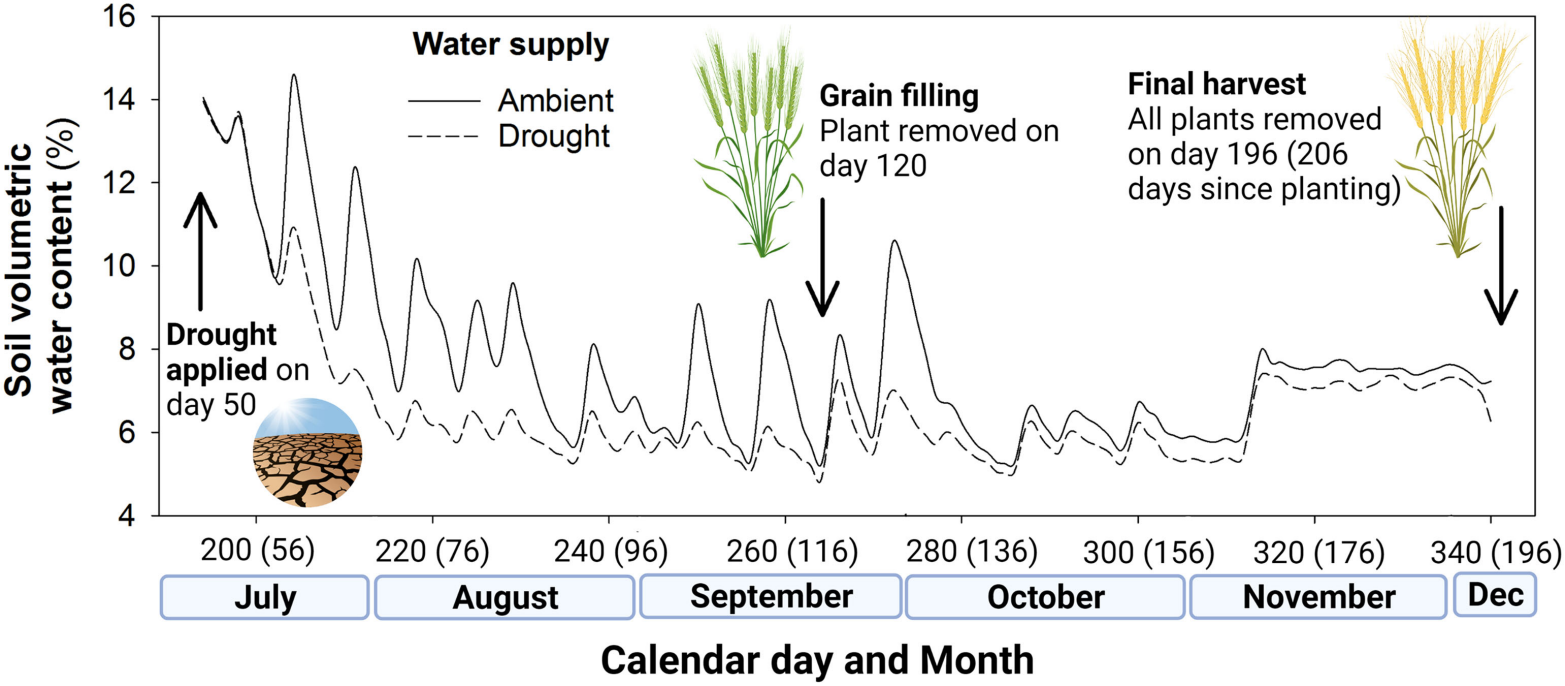
Soil volumetric water content (%) of the ambient well-watered plots and drought (50% rainfall reduction) treatment plots in the rain exclusion shelters, including key events of drought treatment application and the two harvests during the field experiment. Days since seedling emergence given in parentheses.

The 12 shelters were assigned at random to one of two irrigation regimes: (1) ambient conditions (400 mm water based on a predicted growing season of 240 days; 28,000 mL in total) or (2) drought conditions (200 mm water per growing season; 14,000 mL in total). This was calculated on the basis that 1 mm of rainfall per 1 m^2^ equates to 1,000 mL, so 400 mm of rainfall equals 400,000 mL per m^2^. Scaled for our pot size (0.07 m^2^) this is 28,000 mL. These irrigation regimes were applied from day 50, with the drought shelters receiving 50% of the water of the ambient rainfall shelters, during each irrigation event. The volumetric water content of the soil from shelters receiving ambient and drought levels of irrigation is shown in **Figure 1**. In total, 24,500 mL and 12,250 mL was delivered to ambient and drought pots over 210 days as 18 events, equivalent to growing season rainfall (GSR) of 400 mm ha^-1^ and 200 mm ha^-1^, respectively.

In summary, six shelters supplied ambient irrigation and six shelters supplied drought levels (50%) of irrigation with each shelter containing four pots, each with one of four levels of Si supplementation (including one with zero supplementation). Si treatments were assigned at random within each shelter. The factorial design therefore comprised two irrigation regimes × six shelters × four Si treatments giving 48 pots in total and six replicates of each irrigation–Si treatment combination.

### Leaf gas exchange measurements

Net photosynthetic rate (*A*), stomatal conductance (g_s_), and transpiration rate (E) were recorded on flag leaves using a portable infra-red gas analyser (LI-6400XT, Li-COR, USA). One plant from each of the 48 pots was selected at random and the main tiller tagged before measurement; gas exchange measurements were conducted on the first fully expanded leaf in mid-day (10:00 am to 2:00 pm). The RH of the reference air was fixed at 65–70%. The CO_2_ concentration of the reference air entering the leaf chamber was adjusted with a CO_2_ mixer control unit keeping the CO_2_ concentration of the reference air at *ca.* 420 ppm with a constant flow rate of 500 μmol s^−1^ and a light-saturating photon flux density at 1000 μmol m^−2^ s^−1^ supplied by blue and red light-emitting diodes. Instantaneous leaf-level water use efficiency (WUE*_i_*) was calculated (*A*/E). Measurements were taken on two occasions (1–2 August and 7–8 September 2021).

### First harvest - plant biomass and elemental chemistry

When plants entered grain-filling (early milk) stage (day 120), one plant from each pot was removed using a 25 mm diameter soil corer inserted to a depth of 300 mm; roots were separated from the soil using dry sieving through a 2 mm aperture sieve. This sampling strategy provided an indicative rather than absolute value for root biomass, since not all root material could be retrieved *via* soil cores. Tillers were counted. Plant material was then oven-dried and dry mass quantified before measuring Si, and C and N concentrations. Leaf Si concentrations were determined using approximately 100 mg of ground plant material placed into a small mass holder (PANalytical, Malvern, UK), and then analysed with an X-ray fluorescence spectrometer (Epsilon 3^x^, PANalytical, Malvern, UK), using the procedure and certified reference material described in Hiltpold et al. (2017). This method was based on the approach of Reidinger et al. (2012). We determined leaf N and C concentrations using an elemental analyser (FLASH EA 1112 Series CHN analyser, Thermo-Finnigan, Waltham, MA, USA). Replacement soil was backfilled into the hole.

### Final harvest and yield measurements

All remaining plants were harvested when plants developed to grain production (ripening) stage. After quantifying the number of tillers, shoots were oven-dried and shoot samples taken to quantify Si, C and N concentrations (as above). Dry shoot mass and grain yield were assessed. Spikes (ears) were separated from the plant, oven-dried and weighed. A single-head benchtop thresher (Precision Machine Company, Nebraska, USA) was used to separate the grain from chaff. The total amount of grain from each plant was weighed and then counted using an automatic seed counter (Argus Pacific, New Delhi, India). The 1000-grain weight and grain yield per m^2^ (converted to tonnes per hectare) and the Harvest Index (grain/shoot mass) was also calculated.

### Statistical analysis

Plant traits and responses were averaged for all plants in individual pots to give a ‘per plant’ value. Similarly, an average was taken across the two sampling events where repeated measurements were made. These responses were then analysed using two-way ANOVAs with Si supplementation, water supply and their interaction (Si × water supply) included as fixed terms. Log_10_ transformations were applied to g_s_ and WUE*_i_* data, and square root transformation for root biomass, prior to analysis to meet assumptions of normality and homogeneity of variances. Contrast analysis (see Konietschke et al., 2013) was applied to determine statistical differences between plants without Si supplementation and plants receiving low, medium, and high supplementation of Si (representing field application of Agrisilica at the rate of 150, 300 and 450 kg ha^-1^ or Si supplementation of 39, 78 and 117 kg ha^-1^) under each water supply regime. Contrast analysis was also used to explore which Si supplementation rate was required to change plant traits in drought ‘stressed’ plants to levels comparable with ‘unstressed’ plants receiving ambient levels of water supply. All analyses were conducted in the R statistical package or Genstat (version 18, VSN International, Hemel Hempstead, UK).

## Results

### Plant growth

**Figure 2:**
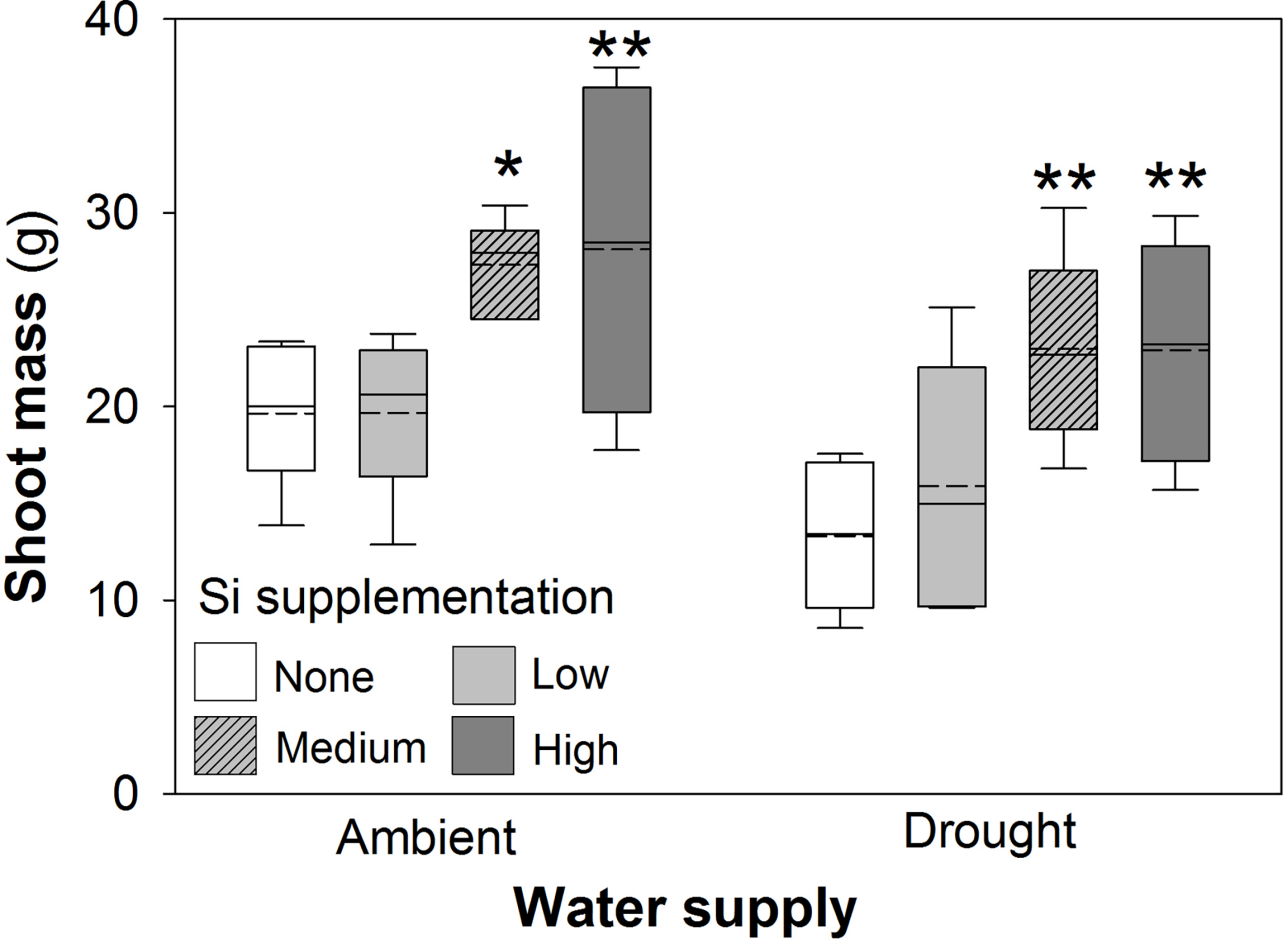
Shoot mass of plants grown with Si supplementation (low, medium and high; 39, 78 and 117 kg ha^-1^) compared to plants without Si supplementation (none) under ambient and drought conditions. Dashed lines represent mean values; solid lines depict the inclusive median (N = 6). The interquartile range is shown. Statistically significant differences between Si supplemented plants and non-supplemented plants indicated *P < 0.05 and **P < 0.01.

Drought conditions significantly reduced shoot mass (–21%; averaged across Si treatments and hereafter) and the number of tillers (–23%) on each plant, whereas Si supplementation increased shoot mass by up to 43% in ambient conditions and 72% in drought conditions (**Figure 2**; **Table 1**) and number of tillers by up to 21% and 36% in ambient and drought plots, respectively (**Table 2**). Root biomass sampling from soil cores suggested that root biomass (results not shown) was negatively impacted by drought (F_1,40_ = 6.67, *P* = 0.014) but unaffected by Si supplementation (F_3,40_ = 0.67, *P* = 0.575). The positive impacts of Si on shoot biomass were seen in medium and high Si supplementation rates under both water supply regimes (**Figure 2**). In terms of alleviating drought stress, this was achieved at low supplementation rates for shoot mass and medium supplementation rates for tiller number (**Table 3**).

**Table 1:** Results of ANOVA analysis of plant trait responses to Si supplementation and water supply, and their interactive influence on these responses.

Plant trait	Figure	Statistical analysis					
		Si		Water supply		Si × Water supply	
		F_3,40_	*P*	F_1,40_	*P*	F_3,40_	*P*
Shoot mass	2	**9.97**	**<0.001**	**10.63**	**0.002**	0.14	0.938
Shoot Si content	3A	**6.72**	**<0.001**	**48.12**	**<0.001**	0.84	0.481
Shoot N content	3B	**8.35**	**<0.001**	**11.44**	**0.002**	0.15	0.930
Shoot C content	4	**7.22**	**<0.001**	**10.78**	**0.002**	0.18	0.911
Grain mass	5	**4.66**	**0.007**	**6.78**	**0.013**	0.29	0.832
Water use efficiency (WUE*_i_*)^1^	6	1.84	0.155	**15.51**	**<0.001**	2.21	0.102
WUE*_i_* ^1^ in drought plants only		**3.53^2^**	**0.034^2^**	–	–	–	–

Statistically significant (P <0.05) results highlighted in bold.

^1^Log_10_ transformation applied. ^2^Degrees of freedom reduced to F_3,20_.

**Table 2:** Responses of plants grown with Si supplementation (low, medium and high; 39, 78 and 117 kg ha^-1^) compared to plants without Si supplementation (none) under ambient and drought conditions.

Si supplementation	Number of tillers (per plant)		Root mass (g)^4^		Shoot Si concentration (% dry mass)		Shoot N concentration (% dry mass)		Shoot C concentration (% dry mass)	
	Ambient	Drought	Ambient	Drought	Ambient	Drought	Ambient	Drought	Ambient	Drought
None	10.04 ± 0.84	6.88 ± 0.67	0.77 ± 0.11	0.35 ± 0.05	0.63 ± 0.03	0.62 ± 0.02	1.13 ± 0.05	1.14 ± 0.07	43.81 ± 0.32	44.05 ± 0.16
Low	9.73 ± 0.61	7.94 ± 0.74	0.57 ± 0.10	0.39 ± 0.07	0.63 ± 0.01	0.61 ± 0.06	1.17 ± 0.10	1.11 ± 0.08	44.09 ± 0.38	43.77 ± 0.14
Medium	10.98 ± 0.41	8.93* ± 0.31	0.62 ± 0.06	0.50 ± 0.04	0.65 ± 0.02	0.57 ± 0.03	1.11 ± 0.09	1.10 ± 0.06	44.10 ± 0.31	43.99 ± 0.30
High	12.17* ± 0.77	9.38* ± 0.71	0.66 ± 0.11	0.68 ± 0.24	0.65 ± 0.02	0.59 ± 0.03	1.15 ± 0.10	1.07 ± 0.05	44.16 ± 0.30	43.90 ± 0.30
***Statistical Analysis***										
	F	*P*	F	*P*	F	*P*	F	*P*	F	*P*
Si^1^	**5.20**	**0.004**	0.95	0.42	0.13	0.944	0.09	0.966	0.10	0.961
Water supply^2^	**27.98**	**<0.001**	**4.75**	**0.035**	**4.76**	**0.035**	0.41	0.527	0.31	0.581
Si × Water supply^3^	0.48	0.70	1.27	0.299	0.65	0.590	0.14	0.937	0.37	0.773

Mean standard ± error shown (N= 6). Statistical test results summarised for Si treatment, water supply and their interaction with statistically significant factors indicated in bold. Statistically significant differences between Si supplemented plants and non-supplemented plants indicated * (P < 0.05).

Degrees of freedom: ^1^ F_3,40_; ^2^ F_1,40_; ^3^ F_3,40_; ^4^ Root mass-root transformation applied.

**Table 3:** Results of contrast analysis for plant traits showing a significant response to Si supplementation.

Plant trait	Figure/Table	Ambient plants (non-supplemented) versus:							
		Drought plants (non-supplemented)		Drought plants (low Si supplementation)		Drought plants (medium Si supplementation)		Drought plants (high Si supplementation)	
		F	*P*	F	*P*	F	*P*	F	*P*
Tiller number	Table 2	**11.67**	**< 0.001**	**5.14**	**0.029**	1.44	0.237	0.52	0.475
Shoot mass	2	**4.41**	**0.042**	1.55	0.221	1.21	0.277	1.17	0.287
Shoot Si content	3A	**9.41**	**0.004**	**6.78**	**0.013**	2.36	0.132	1.30	0.262
Shoot N content	3B	3.90	# 0.055	1.27	0.266	0.79	0.380	0.87	0.355
Shoot C content	4	**4.97**	**0.031**	1.53	0.223	0.55	0.463	0.79	0.378
Grain mass	5	3.07	# 0.087	0.81	0.374	0.01	0.913	0.61	0.440
WUE*_i_*	6	0.01	0.975	**5.46**	**0.025**	3.20	# 0.081	**11.81**	**0.001**

The contrasts are between ‘unstressed’ plants receiving ambient water supply without Si supplementation (non-supplemented) and ‘drought’ stressed plants grown under drought conditions either without Si supplementation or one of three levels of Si supplementation (low, medium or high). Statistically significant (P < 0.05) results shown in bold; results marked # indicate P < 0.10. Unshaded values indicate plant traits that were significantly lower under drought conditions; values shaded grey indicate that the plant trait had statistical parity with ‘unstressed’ plants and those shown in black indicate the plant trait was significantly higher than in ‘unstressed plants’.

### Elemental chemistry

**Figure 3:**
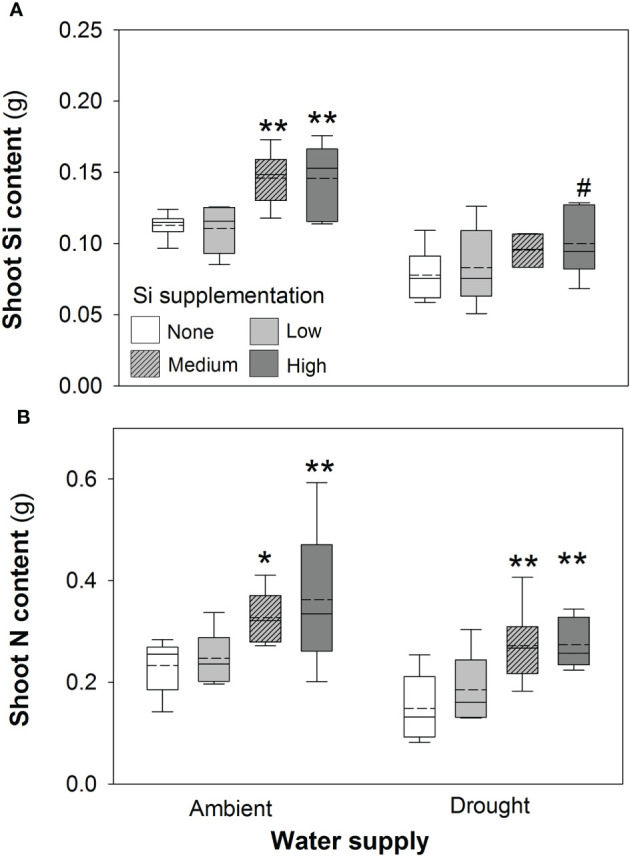
Shoot **(A)** Si and **(B)** N content of plants grown with Si supplementation (low, medium and high; 39, 78 and 117 kg ha^-1^) compared to plants without Si supplementation (none) under ambient and drought conditions (details as in **Figure 2**; N = 6). Statistically significant differences between Si supplemented plants and non-supplemented plants are indicated as *P < 0.05 and **P < 0.01. Marginal non-significance (*P* < 0.10) at the 95% confidence interval indicated #.

Based on tissue *concentrations* (% dry mass) of Si, C and N the total plant *content* of these elements was also calculated (biomass × [concentration/100]. Drought significantly reduced concentrations of Si in the shoots by *ca*. 7% (**Table 2**). However, Si supplementation did not impact shoot Si concentrations, which remained consistent across treatments (0.63–0.65%; **Table 2**) but substantially increased Si content in plant shoots (up to 29%) under ambient water supply conditions (**Table 1**) *via* increased shoot biomass. This occurred with medium and high Si supplementation rates and not at the lower rate of Si supplementation (**Figure 3A**). While the Si content of plants under drought regimes rose by a similar extent (+28%) in plants supplied with the highest Si supplementation rate relative to non-supplemented plants (**Figure 3A**), this narrowly missed statistical significance (*P* = 0.061) at a 95% confidence interval. The larger increase in Si content under ambient conditions compared to drought conditions reflects the fact that Si concentrations were similar in the former, but negatively impacted by the latter, despite Si supplementation increasing shoot mass to similar extents.

Contrary to our hypothesis, drought conditions did not affect shoot N concentrations (**Table 2**) although drought resulted in substantially lower N content in the plants (**Figure 3B**; **Table 1**) because of reduced shoot biomass production. Si supplementation at medium and high rates resulted in higher N content of plants (**Figure 3B**; **Table 1**) due to higher plant mass production but had no impact on shoot N concentrations (**Table 2**).

**Figure 4:**
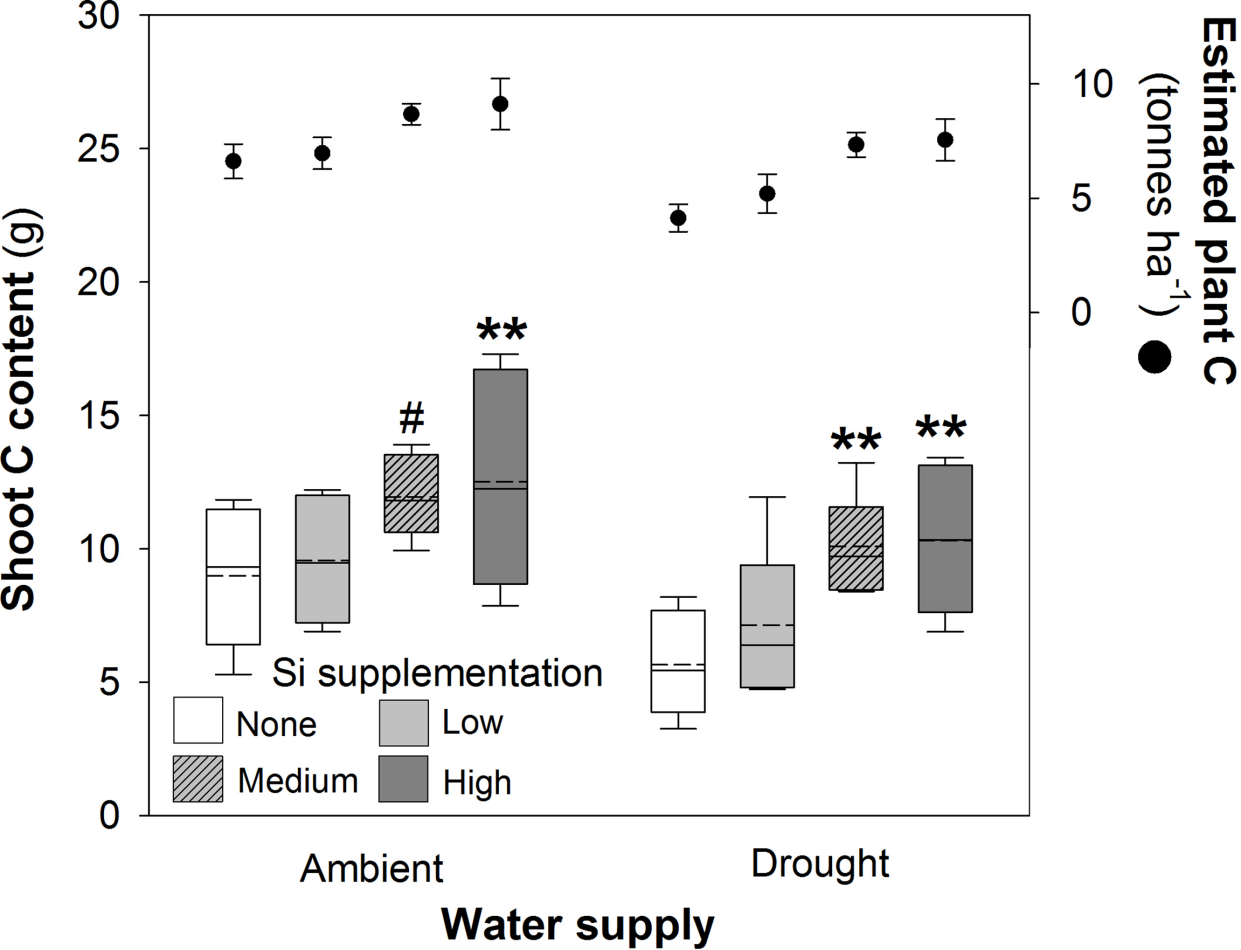
Shoot C content of plants grown with Si supplementation (low, medium and high; 39, 78 and 117 kg ha^-1^) compared to plants without Si supplementation (none) under ambient and drought conditions (details as in **Figure 2**; N = 6). Statistically significant differences between Si supplemented plants and non-supplemented plants indicated **P < 0.01. Marginal non-significance (*P* < 0.10) at the 95% confidence interval indicated #. The estimated total C content of plants per hectare is shown for comparison (mean ± standard error plotted).

Shoot C concentrations were unaffected by water supply or Si supplementation (**Table 2**), but their respective negative and positive impacts on plant mass production resulted in reductions (–23%) and increases (39% and 83% under ambient and drought conditions, respectively) in shoot C content (**Figure 4**; **Table 1**). Increases in shoot C content under ambient conditions were seen most strongly under high Si supplementation rates with smaller increases under medium supplementation rates that narrowly missed statistical significance (*P* = 0.054; Fig 4). Extrapolating linearly to the larger scale based on our sowing rates, Si supplementation at the highest rates would increase C content in shoots from 6.4 to 8.9 tonnes C ha^-1^ under ambient water conditions and 4.0 to 7.4 tonnes C ha^-1^ under drought conditions. This could be further increased if C content of the roots was also taken into consideration. Based on the initial measurements of root mass taken with soil cores, which did not recover all root mass, the total C content of plants would rise from 6.6 to 9.1 tonnes C ha^-1^ under ambient conditions and 4.1 to 7.5 tonnes C ha^-1^ under drought conditions (**Figure 4**).

Comparing control plants receiving ambient levels of water without Si supplementation with the droughted plants, indicated that medium and high levels of Si supplementation was sufficient for the drought affected plants to achieve comparable (statistically indistinguishable) levels of Si content (**Table 3**). This was achieved with low levels, and above, of Si supplementation for shoot N and C content (**Table 3**).

### Grain mass and yield

**Figure 5:**
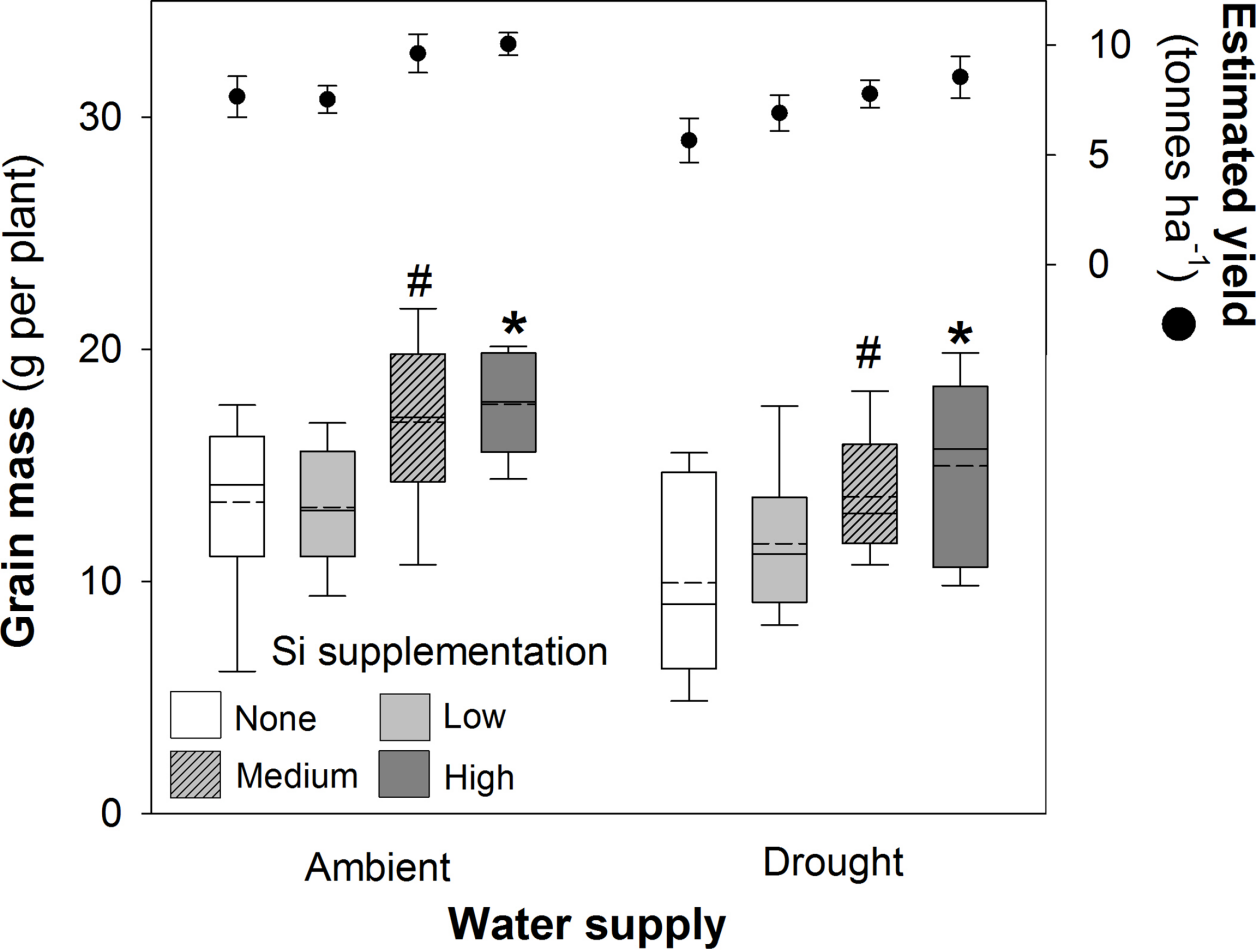
Grain mass of plants grown with Si supplementation (low, medium and high; 39, 78 and 117 kg ha^-1^) compared to plants without Si supplementation (none) under ambient and drought conditions (details as in **Figure 2**; N = 6). Statistically significant differences between Si supplemented plants and non-supplemented plants indicated as *P < 0.05. Marginal non-significance (*P* < 0.10) at the 95% confidence interval indicated #. The estimated grain yield per hectare is shown for comparison (mean ± standard error plotted).

Si supplementation under both watering regimes increased grain mass (**Table 1**), although this was statistically significant only at the highest supplementation rates (**Figure 5**). As hypothesised, drought reduced (–18%) grain production in plants (**Figure 5**; **Table 1**). However, the high Si supplementation rate in drought plants generated similar grain yield as plants grown under ambient water supply without Si supplementation (**Table 3**). Droughted plants receiving low and medium Si supplementation rates were statistically indistinguishable from non-supplemented plants receiving ambient levels of water when the contrast analysis was conducted (**Table 3**).

Based on the current sowing rates, we estimate an increase in grain yield from 7.7 to 10.1 tonnes ha^-1^ (39%) under ambient water conditions and 5.7 to 8.5 tonnes ha^-1^ (49%) under drought conditions (**Figure 5**). As discussed below, this is likely to be an experimentally inflated estimate. The 1000-grain mass was unaffected by Si supplementation reflecting that more grain, rather than larger grain, was being produced when plants received Si supplementation; drought reduced this yield index by *ca*. 5% (**Table 4**). The Harvest Index (ratio of grain yield to whole plant mass) was unaffected by drought or Si supplementation indicating that these treatments did not influence relative allocation of resources to vegetative and reproductive (i.e. grain production) growth (**Table 4**).

**Table 4:** Yield and leaf gas exchange responses to Si supplementation at three rates (low, medium and high) compared to non-supplemented plants (none) averaged across two sampling events.

Si supplemenation	1000 grain mass (g)		Harvest index		Rates of photosynthesis (µmol CO_2_ m^-2^ s^-1^)		Stomatal conductance^4^ (mmol H_2_O m^-2^ s^-1^)		Transpiration rates (mmol H_2_O m^-2^ s^-1^)	
	Ambient	Drought	Ambient	Drought	Ambient	Drought	Ambient	Drought	Ambient	Drought
None	48.49 ± 0.45	45.26 ± 1.01	0.67 ± 0.05	0.72 ± 0.05	16.76 ± 1.41	16.84 ± 1.32	0.23 ± 0.03	0.17 ± 0.03	2.53 ± 0.37	2.43 ± 0.21
Low	48.48 ± 0.79	45.87 ± 0.44	0.68 ± 0.06	0.78 ± 0.08	18.81 ± 0.98	15.48 ± 1.19	0.27 ± 0.03	0.15 ± 0.03	2.96 ± 0.23	1.81 ± 0.24
Medium	47.75 ± 0.38	46.88* ± 0.22	0.61 ± 0.04	0.61 ± 0.05	17.74 ± 1.31	15.82 ± 1.20	0.37 ± 0.08	0.16 ± 0.03	2.73 ± 0.36	1.89 ± 0.17
High	48.49 ± 0.28	46.54 ± 0.39	0.66 ± 0.07	0.66 ± 0.04	18.85 ± 0.85	14.81 ± 1.29	0.29 ± 0.03	0.13 ± 0.02	2.99 ± 0.37	1.55 ± 0.33*
*Statistical analysis*										
	F	*P*	F	*P*	F	*P*	F	*P*	F	*P*
Si^1^	0.47	0.702	1.67	0.188	0.04	0.989	0.65	0.589	0.19	0.901
Water supply^2^	**30.41**	**<0.001**	0.75	0.392	**7.28**	**0.010**	**34.46**	**<0.001**	**18.12**	**<0.001**
Si × Water supply^3^	1.66	0.190	0.37	0.776	1.13	0.348	1.26	0.302	1.94	0.139

Mean standard ± error shown (N= 6). Statistical test results summarised for Si treatment, water supply and their interaction with statistically significant factors indicated in bold. Statistically significant differences between Si supplemented plants and non-supplemented plants indicated * (P < 0.05).

Degrees of freedom: ^1^ F_3,40_; ^2^ F_1,40_; ^3^ F_3,40_. ^4^Log_10_ transformation applied.

### Leaf gas exchange

**Figure 6:**
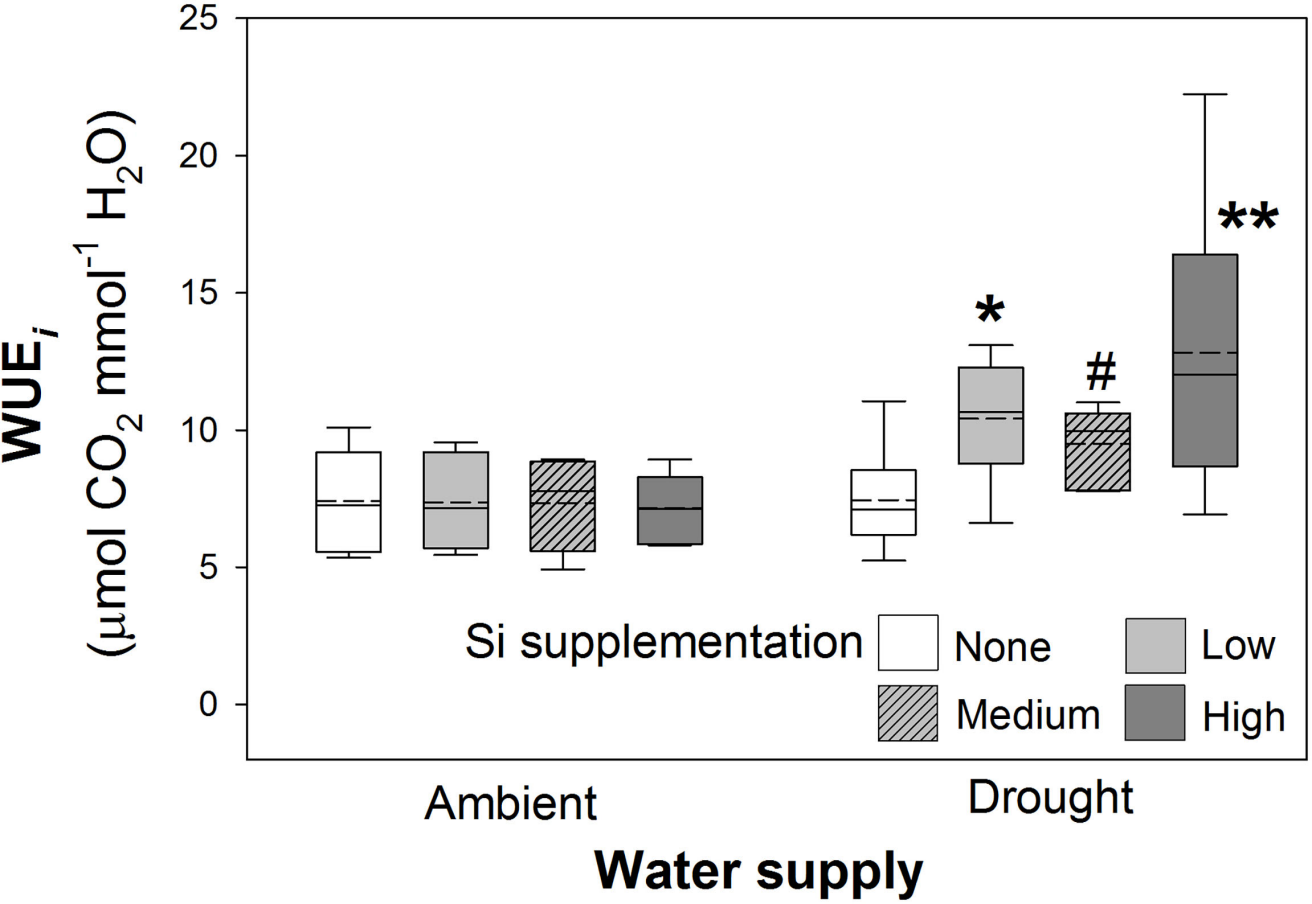
Instantaneous water use efficiency (WUE*_i_*) of plants grown with Si supplementation (low, medium and high; 39, 78 and 117 kg ha^-1^) compared to plants without Si supplementation (none) under ambient and drought conditions (details as in **Figure 2**; N = 6). Statistically significant differences between Si supplemented plants and non-supplemented plants indicated *P < 0.05 and **P < 0.01. Marginal non-significance (*P* < 0.10) at the 95% confidence interval indicated #.

As hypothesised, drought resulted in lower rates of photosynthesis, stomatal conductance, and transpiration rates, which were not significantly affected by Si supplementation (**Table 4**). Instantaneous water use efficiency (WUE*_i_*) significantly increased under drought conditions (**Figure 6**; **Table 1**). Si supplementation significantly increased WUE*_i_* when examined separately for the drought plants (**Table 1**). Using contrast analysis, low and high Si supplementation rates significantly increased WUE*_i_* in drought plants compared to well-watered plants, with medium supplementation rates following the same trend (**Table 3**).

## Discussion

We used a rain-exclusion experimental design to address whether field Si supplementation ameliorated the adverse impacts of drought on wheat biomass production and grain yield. We found that Si supplementation alleviated the negative impact of drought on biomass production and grain yield, which may be due to increased WUE*_i_*.

### Si reduced transpiration rates and increased WUE

Si supplementation has been reported to improve WUE during drought stress (Alzahrani et al., 2018; Ibrahim et al., 2018; Merwad et al., 2018), which can operate *via* increased stomatal conductance, which in turn improves rates of photosynthesis rate (Sonobe et al., 2009; Yin et al., 2014; Wang et al., 2019). In contrast, Si may improve WUE by reducing water loss by lowering, or more tightly regulating, transpiration losses (Agarie et al., 1998; Vandegeer et al., 2021b) which is more consistent with this study. Vandegeer et al. (2021b) demonstrated that reduced transpiration loss was facilitated when Si was deposited in stomatal guard cells and as a sub-cuticular layer. This was associated with a 28% and 23% reduction in stomatal and cuticular conductance, respectively, compared to plants with low Si. Crucially, when abscisic acid (ABA) was applied to promote stomatal closure, plants with silicified guard cells had 19% smaller stomatal apertures and an increased efflux of guard cell K^+^ ions. Hence, Si supplemented plants had increased sensitivity to ABA, which led to more rapid stomatal closure (Vandegeer et al., 2021b). Greater stomatal sensitivity improves WUE by increasing CO_2_ assimilation and limiting water loss (Lawson and Matthews, 2020), which suggests one mechanism that could be facilitated by Si to alleviate drought stress in plants.

It has been shown that improved WUE due to Si alleviates drought stress in wheat (Maghsoudi et al., 2016; Alzahrani et al., 2018), although additional mechanisms such as reductions in oxidative damage and anti-oxidative enzyme activity may also be important (Ma et al., 2016; Alzahrani et al., 2018). Unlike Alzahrani et al. (2018) and Maghsoudi et al. (2016), we did not observe Si-induced changes in rates of photosynthesis and stomatal conductance in wheat. Si may increase rates of photosynthesis because silicification promotes more erect plant growth that facilitates higher light interception (Ma et al., 1989; Quigley and Anderson, 2014). However, in our study with wheat growing in high light environments, the plants may not have been light-limited.

### Increased C capture and increased yield

We expected that Si supplementation would increase the concentration of Si in plant tissues but shoot Si concentration remained stable across all Si supplementation rates. However, Si supplemented plants exhibited greater mass production than non-supplemented plants, which resulted in greater Si content in Si plants than non-supplemented plants. Where Si concentrations are reported to increase in pot experiments, usually with repeated Si supply, this often corresponds with a decrease in C concentrations (Rowe et al., 2020; Johnson et al., 2022). This may simply be due to stoichiometric dilution whereby an increase in Si necessitates lower levels of other constituents, with C being the most abundant and therefore most likely to decline (Quigley et al., 2020). Alternatively, it may reflect plants using Si as a ‘metabolically cheaper’ substitute in lieu of defensive (e.g. phenolics) (Cooke and Leishman, 2012) or structural (e.g. cellulose) C-based compounds (Schoelynck et al., 2010).

In our study, we saw that C concentrations remained stable, but total C content of plants rose substantially, driven primarily by increased plant biomass production, with Si supplementation increasing content from 6.4 to 8.9 tonnes C ha^-1^ (+39%) under ambient conditions and 4.0 to 7.4 tonnes C ha^-1^ (+82%) under drought conditions. A potential benefit of increased C content in Si-supplemented wheat plants may be increased C capture and sequestration, particularly if C was transferred to soil C pools (Beerling et al., 2018). This is highly speculative, of course, but a recent assessment suggested that rock weathering using silicate rocks could deliver net atmospheric CO_2_ removal of 6–30 Mt CO_2_ yr^−1^ for the UK by 2050 (Kantzas et al., 2022).

### Options for Si supplementation

One significant environmental constraint on Si uptake is native soil Si availability (Gocke et al., 2013). This can be addressed in part through Si fertilizers, including slags from steel making and blast furnaces (which contain calcium silicate, Ca_2_SiO_4_), manufactured forms including sodium silicate (Na_2_SiO_3_) or potassium silicate (K_2_SiO_3_) (Haynes, 2017), and commercially available naturally occurring forms, such as wollastonite, diatomaceous earth and amorphous silica (used in the current study) (Haynes, 2017; Zellner et al., 2021).

A recent analysis suggested that although the costs and benefits of Si supplementation will depend on a large number of factors, such as crop type, yield per hectare, anticipated yield gain and production costs, some Si fertilisers could be economically feasible and environmentally sustainable (Thorne et al., 2020). Based on our grain yield results in small experimental plots, we estimated that Si supplementation would increase yield from 7.7 to 10.1 tonnes ha^-1^ (+39%) under ambient, well-watered conditions and 5.7 to 8.5 tonnes ha^-1^ (+49%) under drought conditions. While yields of this magnitude are possible for European wheat and Australian wheat receiving irrigation or high levels of precipitation (*ca*. 8.5 tonnes ha^-1^) (French and Schultz, 1984), the median yield for dryland Australia is 2.4 tonnes ha^-1^ (Wheatcast, 2022), so our experimental conditions may have favoured higher yields than might be realised commercially. This is relatively common for wheat breeding trials conducted under semi-field conditions because of better maintenance, more open canopies and less crowding from surrounding plants (J. Hull, personal communication). Nonetheless, as a rough estimate based on a farmgate price of A$359 per tonne of grain and an estimated cost of A$600 per tonne of Si fertilizer (Agrisilica) (B. Cairns, personal communication), this would represent an increase in profitability of A$592 and A$735 per hectare for ambient and drought scenarios, respectively. A proportionate scaled-down increase might reasonably be expected. Moreover, the 50% reduction in irrigation in the current study would represent a water saving of 1.75 ML ha^-1^. While water prices vary significantly, assuming a cost of A$80 per ML, then an additional saving of A$134 per ha might be achieved, potentially rising to $262 per ha if a higher water cost (A$150 per ML) was levied, as is common during water scarcity.

## Conclusions

This rain-exclusion study has established that Si supplementation had beneficial impacts on wheat biomass production and grain yield such that the negative impacts of drought on these traits could be negated. Si supplementation using environmentally safe Si fertilisers, such as amorphous silica which typically has higher Si content than other fertilisers and fewer contaminants, could provide a useful tool for drought mitigation strategies.

## Data availability statement

The raw data supporting the conclusions of this article will be made available by the authors, without undue reservation.

## Author contributions

SJ, Z-HC and DT conceived the work and obtained funding for the research. RR established the experiments, conducted the experimental procedures and processed plant and soil material. SJ collated and analysed data and produced the first draft of the manuscript. All authors contributed to the article and agree to its findings and publication. All authors approved the submitted version.

## Acknowledgments

We thank Dr. Meiqin Lu at AGT Breeding for providing the wheat seeds and Pushpinder (Simmy) Matta for conducting the C and N analysis. This research was funded by a Western Sydney University (WSU) Industry Partnership grant awarded to SJ, DT and Z-HC, funding for which was provided from WSU and Agripower Australia Ltd. SJ received support from an Australian Research Council Future Fellowship (FT170100342). 

## Conflict of interest

The research was partially funded by Agripower Australia Ltd who provided the product used for the silicon supplementation. This was regulated under contract issued by Western Sydney University as part of an Industry Partnership Grant with payment being independent of research findings. Agripower Australia provided voluntary feedback on the manuscript but did not attempt to influence the interpretations or conclusions of the work.

The authors declare that the research was conducted in the absence of any commercial or financial relationships that could be construed as a potential conflict of interest.

## Publisher’s note

All claims expressed in this article are solely those of the authors and do not necessarily represent those of their affiliated organizations, or those of the publisher, the editors and the reviewers. Any product that may be evaluated in this article, or claim that may be made by its manufacturer, is not guaranteed or endorsed by the publisher.
