# Supplementary material for: Field application of silicon alleviates drought stress and improves water use efficiency in wheat
Source: Front Plant Sci. 2022 Nov 9;13:1030620. doi: 10.3389/fpls.2022.1030620 (PMC9682199; doi:10.3389/fpls.2022.1030620)
Supplement: Supplementary file 1 [file Table_1.docx]

**Supplemental Material**

**Table S1**. Soil chemical composition (mean values shown)

| **Soil parameter** (and units) | | **Concentration** |
| --- | --- | --- |
| Soluble Calcium (mg/kg) | | 590 |
| Soluble Magnesium (mg/kg) | | 153 |
| Soluble Potassium (mg/kg) | | 171 |
| Soluble Phosphorus (mg/kg) | | 1.3 |
| Phosphorus (mg/kg P) | Bray 1 | 4.4 |
|  | Colwell | 18 |
|  | Bray 2 | 7.1 |
| Nitrate Nitrogen (mg/kg N) | | 343 |
| Ammonium Nitrogen (mg/kg N) | | 83 |
| Sulfur (mg/kg S) | | 17 |
| pH | | 6.16 |
| Exchangeable Calcium | (cmol_+_/kg) | 3.9 |
|  | (kg/ha) | 1,734 |
|  | (mg/kg) | 774 |
| Exchangeable Magnesium | (cmol_+_/kg) | 1.4 |
|  | (kg/ha) | 382 |
|  | (mg/kg) | 171 |
| Exchangeable Potassium | (cmol_+_/kg) | 0.56 |
|  | (kg/ha) | 487 |
|  | (mg/kg) | 217 |
| Exchangeable Sodium | (cmol_+_/kg) | 0.22 |
|  | (kg/ha) | 111 |
|  | (mg/kg) | 50 |
| Exchangeable Aluminium | (cmol_+_/kg) | 0.01 |
|  | (kg/ha) | 2.4 |
|  | (mg/kg) | 1.1 |
| Exchangeable Hydrogen | (cmol_+_/kg) | 0.03 |
|  | (kg/ha) | <1 |
|  | (mg/kg) | <1 |
| Effective Cation Exchange Capacity (ECEC) (cmol_+_/kg) | | 6.1 |
| Calcium (%) | | 64 |
| Magnesium (%) | | 23 |
| Potassium (%) | | 9.1 |
| Sodium - ESP (%) | | 3.5 |
| Aluminium (%) | | 0.19 |
| Hydrogen (%) | | 0.49 |
| Calcium/Magnesium Ratio | | 2.8 |
| Zinc (mg/kg) | | 1.5 |
| Manganese (mg/kg) | | 454 |
| Iron (mg/kg) | | 33 |
| Copper (mg/kg) | | 0.54 |
| Boron (mg/kg) | | 0.63 |
| Silicon (mg/kg Si) | | 22 |
| Total Carbon (%) | | 0.85 |
| Total Nitrogen (%) | | 0.12 |
